# Evaluating the role of the smartjournal digital intervention in improving oral hygiene among nursing home residents: a cluster randomised trial

**DOI:** 10.1186/s12903-025-06994-0

**Published:** 2025-10-10

**Authors:** Enes Karamehmedovic, Lene Elisabeth Myhren, Knut Helge Midtbø Jensen, Ewa Joanna Rodakowska, Anne-Kristine Nordrehaug Åstrøm, Elisabeth Lind Melbye

**Affiliations:** 1 Department of Clinical Dentistry, University of Bergen, Bergen, Norway; 2 Oral Health Centre of Expertise – Rogaland, Stavanger, Norway; 3 Oral Health Centre of Expertise – Vestland, Stavanger, Norway; 4 Department of Clinical Dentistry – Cariology section, University of Bergen, Bergen, Norway; 5 Faculty of Health Sciences, Department of Public Health, University of Stavanger, Stavanger, Norway

**Keywords:** Oral health, Older adults, Nursing home, Digital intervention, Long-term care, Cluster randomised trial, Public health

## Abstract

**Background:**

The increasing ageing population has led to increased incidence of chronic conditions, including oral diseases, which are prevalent and overlooked in older adults, especially those in institutional care. Poor oral health is linked to systemic disease, reduced quality of life, and challenges in caregiving, particularly among individuals with dementia who often resist assistance. Barriers related to oral care reported by caregivers include a lack of training, time, and documentation systems. Current interventions largely focus on staff education, with limited and inconsistent outcomes. To support caregivers, a digital tool, the SmartJournal, has been developed to improve oral care through documentation, assessment and education. The aim of the present study was to evaluate the effectiveness of the use of SmartJournal for improving oral hygiene among nursing home residents.

**Methods:**

A pragmatic parallel-group cluster randomised trial was conducted in 12 nursing homes in Rogaland County, Norway. The trial lasted for nine months, with clinical assessments at baseline and at the 3-month and 9-month follow-ups. Randomisation occurred at the nursing home level via simple randomisation. The mucosal-plaque score (MPS) was used as the primary outcome measure. A generalised linear mixed-effects model (GLMM) was used to evaluate the effect of the intervention, adjusted for participant characteristics and clustering, following the intention-to-treat (ITT) approach.

**Results:**

The analysis revealed no significant difference in oral hygiene between the groups at baseline (*p* = 0.455) or at the second follow-up (*p* = 0.292). Within-group analysis revealed a significant improvement during the study in the intervention group (*p* = 0.047).

**Conclusions:**

The SmartJournal intervention led to greater improvement in the oral hygiene score, but the difference between the groups was not statistically significant. A larger-scale study is needed to confirm these findings.

**Trial registration:**

NCT05724043. Date: 2023-01-10.

**Supplementary Information:**

The online version contains supplementary material available at 10.1186/s12903-025-06994-0.

## Introduction

 The global trend of population ageing has led to a progressive demographic transformation characterised by a growing percentage of older individuals in modern industrialised societies [1]. In Norway, the population aged 67 and above has increased by 48% since 2000 and is expected to grow by another 54% by 2050 [2]. This ageing of the population also contributes to an increasing burden of chronic diseases [3, 4], posing a future challenge to public health services [5]. Among these diseases, oral diseases are particularly prevalent in older adults [3, 6–8], despite being largely preventable with proper daily oral hygiene [9].

The declining rate of edentulism (no teeth present), which is frequently restored with complex dental prostheses [10], reflects an emphasis on the importance of maintaining good oral health. The burden of poor oral health can be seen in individuals’ physical and psychosocial health and daily life [11]. This can manifest as low self-esteem, chewing difficulties and dental pain, which may alter dietary habits, leading to malnutrition and cognitive decline [6, 7, 12]. Research has shown associations between poor oral health and systemic infections and diseases [13], such as cardiovascular [14] and pulmonary disease [15], dementia [16], and even premature death [11]. Despite its importance, oral health is often overlooked and remains a prevalent concern for older individuals [3, 12, 13], particularly in institutionalised settings [4–6], where unique barriers to upholding good oral health exist [4, 17, 18]. Consequently, care-dependent individuals are notably susceptible to rapid deterioration of oral health [12].

Many long-term care-dependent residents require assistance with daily oral hygiene. However, health-care professionals encounter multiple barriers in providing adequate oral care [17, 18]. These barriers include an absence of specialised oral care training, insufficient knowledge and skills, high workloads and inadequate systems for documenting oral health issues [4, 18–21]. Furthermore, the complexity of dental care has increased, and many individuals with dementia frequently exhibit uncooperative behaviour, refusing assistance with oral hygiene (i.e., care-resistant behaviour; CRB) [18, 22]. A study among Norwegian nursing home residents revealed that more than 50% of those with dementia refused oral care assistance [22]. Kvalheim et al. [23] reported that 25% of healthcare institutions in Norway did not have established oral care procedures, and half of them did not consider oral health issues significant.

A study by Willumsen et al. [22] found that more than 40% of nursing home residents had unacceptable oral hygiene. Subsequent research identified oral health as the most neglected area in Norwegian nursing homes [24], often the first task to be deprioritised due to resource constraints [25]. A prior study reported that many nursing home residents with natural teeth suffer from at least one oral health-related issue [26], with poor oral hygiene being omnipresent among care-dependent older adults [12]. Common problems include xerostomia (dry mouth), gingival bleeding, plaque accumulation, cavities, and mucosal lesions [8, 27].

Previous reviews of interventions targeting the oral health of care-dependent older adults found that strategies largely focused on enhancing caregivers’ oral health knowledge or promoting behavioural changes, rather than directly improving residents’ oral health [11, 28]. While no intervention has demonstrated clearly superior effectiveness [11, 28], significant variability in study outcomes complicates comparisons across interventions [12]. Some studies have explored a more targeted approach. A longitudinal study of an oral hygiene protocol showed minimal improvements in plaque levels [29]. A randomised trial assessing improvements in oral hygiene among nursing home residents reported no significant difference between groups [30] or resulted only in short-term improvement [31]. Additionally, a study focusing on staff education reported no notable findings in improving residents’ oral health outcomes [32].

A systematic review by Cochrane suggested that future interventions should focus on nursing home staff to enhance their competence in delivering oral care, which could lead to better oral health outcomes for residents [28]. Considering the limited number of clinical trials evaluating the effectiveness of preventive oral health-care interventions for care-dependent older adults [12], a key requirement for future trials must be to determine the impact of pragmatic interventions on residents’ oral health [28]. The World Health Organisation has identified digital technology as a potential solution of scalable and sustainable improvements in health outcomes [33]. In this context, digital tools that support daily care practices and information sharing – particularly in nursing home settings – can play a vital role in improving health outcomes.

In response to these challenges, and to address the oral healthcare barriers reported by nursing home caregivers, an interactive and user-friendly digital tool named SmartJournal (SJ) was developed in collaboration with researchers at the Oral Health Centre of Expertise Rogaland, Norway, dental professionals, nursing home caregivers and innovative research and development institutions to support caregivers in maintaining the oral health of care-dependent individuals. It combines procedures for oral health related documentation and assessment, acts as a learning tool for upholding adequate oral health and provides strategies for handling CRB. The tool contains three components designed to support and improve oral health care in nursing home settings: Component 1, “registration of daily oral hygiene routines”. This component provides a system for documenting residents’ oral hygiene routines. It also allows records of any deviations from standard practices, helping caregivers stay informed about potential issues and enabling early intervention. Component 2, “monthly oral health assessment”, offers a monthly check-up system of residents’ oral health. It supports systemic monitoring and helps ensure timely identification of oral health concerns. Component 3, “e-learning (knowledge base)”, an easily accessible knowledge base which offers comprehensive information and practical guidance on oral health and hygiene. It serves as an educational resource for caregivers, supporting learning and consistent care practices. Further details about the tool have been extensively described elsewhere [34, 35].

### Aim

The aim of this study was to evaluate whether the use of SmartJournal by nursing home caregivers improves oral hygiene outcomes among care-dependent nursing home residents.

## Materials and methods

### Study design and randomisation

The study was designed as a pragmatic, parallel-group, three-arm blinded cluster-randomised trial (CRT) aiming to compare the effectiveness of SJ use (intervention) versus augmented care (control) in improving oral hygiene among care-dependent adults in nursing homes. CRT was found to be more practical in the present study, as the intervention was designed to be delivered by a group of people (nursing staff at selected nursing homes) rather than individuals. Furthermore, the results from a CRT increase the generalisability of the findings, as it reflects real-life conditions and includes a representative cohort of the population [36]. In this study, nursing homes were randomly allocated to the intervention or control group. As such, nursing homes were not eligible if they met any of the following exclusion criteria:

1. Specialised nursing homes for mental health disorders or addiction,
2. Nursing homes that offer only short-term stays,
3. Nursing homes that were part of initial testing of SJ,
4. Nursing homes with fewer than 40 long-term residents.

Simple randomisation was used to allocate clusters. A total of 16 nursing homes in Rogaland County, Norway were eligible for participation in the study. The names of all eligible nursing homes were placed in sealed opaque envelopes prepared by the first author (EK). On the basis of power analysis calculations, 12 nursing homes were determined to be necessary for the study. The project coordinator randomly selected 12 envelopes and assigned them to either the intervention or the control group in a 1:1 ratio, with six nursing homes in each group. This approach helped minimise potential differences between the groups and reduced the risk of allocation bias. If a selected nursing home was unable to participate in the study, the project coordinator selected an alternative facility by drawing a new envelope. The outcome of the randomisation process was concealed from everyone except the first author and the project coordinator, ensuring allocation concealment. The CONSORT statement for CRTs was followed for designing, analysing and reporting this study [37] [Additional file 1].

### Participants and procedures

The participants were long-term residents in nursing homes. The exclusion criteria for participation were as follows:

1. Short-term or day attendance only,
2. Residents who verbally or physically opposed examinations,
3. Residents with terminal illness (not expected to survive the 9-month study period).

#### Calibration

Before the clinical examinations, excessive pictorial training was provided for all dental personnel (two dentists and two dental hygienists) who served as data collectors in the trial. For calibration, 42 images of nursing home residents were used to assess inter-rater reliability (IRR) among the data collectors. The following parameters were evaluated: MS, PS and MPS. The intraclass correlation coefficients (ICC_class) and their 95% confidence intervals are presented in Table 1. A two-way mixed-effects model for absolute agreement (ICC_class (A, k)) was used to assess the consistency of ratings across four raters [38, 39]. The ICC_class values for MS, PS and MPS were 0.983, 0.970 and 0.953, respectively. Values above 0.9 indicate excellent reliability, suggesting a high degree of agreement and minimal measurement error [39].

**Table 1:** Results of the ICC_class calculation using absolute agreement, average measures of fixed number of raters (k = 4), and a 2-way mixed-effects model

	Intraclass Correlation Coefficient	95% CI		Value	df1	df2	*P* value
		Lower	Upper				
Mucosal score^a^	0.983	0.973	0.991	62.546	3	168	< 0.001
Plaque score^b^	0.970	0.950	0.984	40.033	3	168	< 0.001
Mucosal-plaque score^c^	0.953	0.924	0.973	22.599	3	168	< 0.001

^a^ICC_class for MS

^b^ICC_class for PS

^c^ICC_class for MPS

#### Sample size and power estimation

**Fig. 1:**
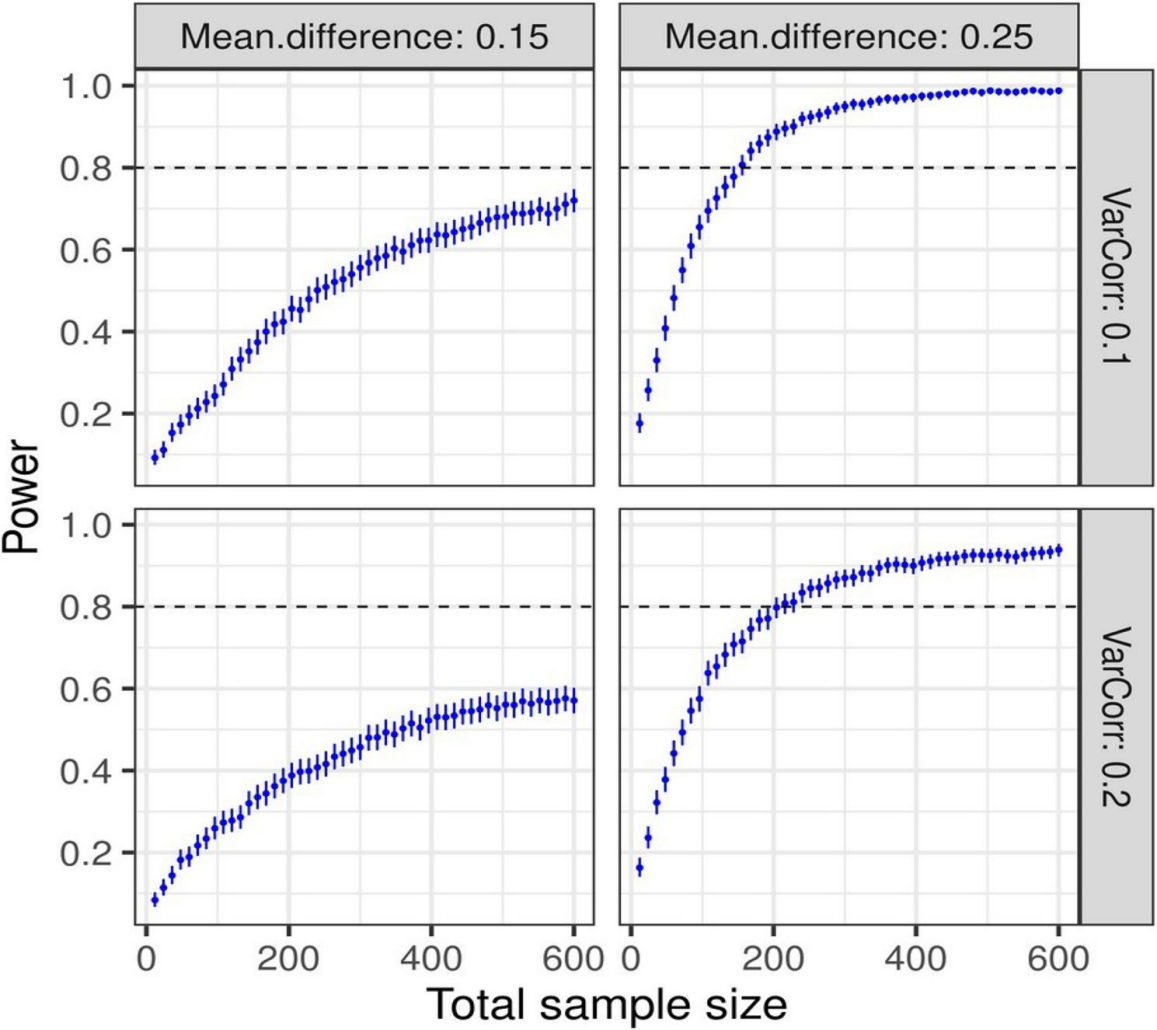
Sample size and power estimation

Power estimations were based on the Monte Carlo simulation for a model comparing the control and intervention groups (Fig. 1). Simulations were done by using the simr package of R [40]. We aimed to detect fixed effect sizes of 0.15 and 0.25 (left and right panels, respectively) and with a cluster-level random effect (nursing homes), measured in variance set to 0.1 or 0.2 (right title bars), with an intracluster correlation coefficient (ICC) = 0.03 or 0.06. Each group included six nursing homes. In the simulations we used the glmer function from the lme4 package of R [41] to specify the model for the simulations. In this model, the response variable was binary: individuals who showed a reduction in MPS from baseline were coded as 1 (success), while those without a reduction were coded as 0 (failure). The results indicated that a total sample size of at least 200 participants was needed to achieve 80% power (0.8), assuming a mean difference in probability of success between groups of no less than 0.25.

#### Recruitment

Recruitment began with contacting selected nursing homes to explore their willingness to participate in the study. Following agreement, members of the research team visited the recruited nursing homes to explain the trial’s details. At each nursing home a few employees were selected to form a “task group” to cooperate with the research team. They were responsible for recruiting individual residents and obtaining informed consent from all study participants – either from the residents or, in cases of reduced decision-making capacity, from their next of kin. An additional file shows a flow chart outlining the recruitment process for both nursing homes and participants [see Additional file 2: Figure S1].

#### Blinding

To protect the randomisation sequence, maintain allocation concealment, and minimise ascertainment bias after allocation, blinding one or more individuals was implemented, as recommended for an CRT [42, 43]. In this study, blinding occurred across three arms. At participant level, the nursing home residents were blinded. At caregiver level, blinding helped reduce performance and ascertainment bias. This included the task group responsible for resident recruitment who was unaware of group assignment, preserving allocation concealment up to the point of intervention and preventing selection bias [42]. At the assessor level, three dental personnel who served as data collectors were fully blinded to group allocation to limit observer bias. Although the first author, who also participated in the data collection, was not blinded, efforts were made to minimise bias by avoiding contact with the nursing homes between recruitment and data collection. Finally, the last blinded arm was the data analyst in charge of analysing the trial results.

#### Intervention and control groups

Nursing homes allocated to the intervention group received in-person and video tutorial on the use of SJ, along with supplies that included iPads preinstalled with SJ, chargers and disinfection wipes. The app was used by nursing home caregivers to support and document residents’ oral hygiene practices, providing prompts, guidance, and monitoring features to reinforce oral care routines. Members of the task group had biweekly meetings during the first three months of the study with the second author (LM) to discuss any potential concerns or questions. Furthermore, the task group were responsible for training new employees. All participants (i.e., participants in both the intervention and the control group) received a care package containing supplies necessary to maintain oral hygiene. As a result, the control group was classified as an augmented care group rather than a standard care group [44]. Given the vulnerability of the study population, study design and ethical considerations outlined in the Ottawa Statement [45], it was deemed unrealistic and unethical to withhold all forms of assistance from participants, even at the cost of potentially reducing the intervention’s effect size. This enhancement also addressed a potential confounder related to the provision of oral care supplies by participants’ next of kin.

#### Clinical examinations

Clinical examinations were conducted by dental professionals immediately before the intervention (baseline, T0) and at the 3-month (T1) and 9-month (T2) follow-ups. Examinations were conducted within the nursing home, either in the resident’s room or in the living room area, wherever the individual felt most comfortable. Visual inspections of the oral cavity were performed using two dental mirrors and a portable light source [31]. Oral hygiene was measured using the mucosal-plaque score (MPS), which evaluates both dental plaque accumulation and oral inflammation [46]. If a resident had prostheses, these were examined for plaque in line with the criteria for plaque score on teeth and reported as such. The following parameters were evaluated: mucosal score (MS) (scores 1–4) and plaque score (PS) (scores 1–4). The criteria for MS were as follows: (1) normal appearance, (2) mild inflammation, (3) moderate inflammation, and (4) severe inflammation, whereas the criteria for PS were as follows: (1) no visible plaque, (2) small amounts of plaque, (3) moderate amount of plaque, and (4) abundant amount of plaque [46]. The sum of MS and PS was labelled MPS (scores 2–8). Clinical examinations also included the assessment of the number of natural teeth, which will not be discussed in this article. Additionally, if clinical examinations revealed signs of oral disease, the residents would be referred to the local dental clinic to ensure timely intervention and mitigate the risk of disease progression.

### Statistical analysis

All analyses, figures and statistical models have been carried out by using R version 4.4.1 for Windows (R Core Team 2024). The primary outcome variable in the study was a dichotomised MPS score following Henriksen et al. [46] by defining acceptable (MPS ≤ 4) and unacceptable/poor (MPS >4) oral hygiene, giving the first and latter binary score of 0 and 1, respectively. The MPS values of the dichotomisation were only based on natural teeth as long as possible. If only dentures were available, the same dichotomisation rule was followed for MPS values derived from dentures. Since the outcome variable is binary and clustered under nursing homes, the application of a generalised linear mixed-effects model (GLMM) for binary data was considered an appropriate analysis technique [47]. To investigate our research aim we used both an intention-to-treat (ITT) approach and a per-protocol (PP) approach [48, 49]. Descriptive baseline characteristics were summarised for participants in the intervention and control groups, as well as those retained at each of the two follow-ups, to enable comparison over time and assess potential attrition bias. Pearson’s chi-square test was used to compare sociodemographic variables and the prevalence of systemic diseases between groups. Statistical significance was set at a p-value < 0.05.

## Results

Table 2 presents the demographic and clinical characteristics of the participants at baseline and at two follow-up points, on the basis of group allocation. Overall, the groups were comparable at all three time points. At baseline, a significantly greater prevalence of diabetes (*p* = 0.042) and dementia (*p* < 0.001) was observed in the intervention group. The difference in the incidence of diabetes was no longer significant at the first (*p* = 0.076) or second (*p* = 0.060) follow-up. In contrast, the prevalence of dementia remained consistently higher in the intervention group across all time points (*p* < 0.001). While no significant baseline difference was found (*p* = 0.061), the prevalence of kidney disease was significantly greater in the intervention group at both the first (*p* = 0.021) and second (*p* = 0.020) follow-ups.

**Table 2:** Demographic and clinical characteristics of participants at baseline, 3-month and 9-month follow-up by study group

		Baseline (T0)			3-months follow-up (T1)			9-months follow-up (T2)		
		Control (*N* = 170) n (%)	Intervention (*N* = 139) n (%)	p-value	Control (*N* = 150) n (%)	Intervention (*N* = 121) n (%)	p-value	Control (*N* = 117) n (%)	Intervention (*N* = 98) n (%)	p-value
Gender				0.396			0.344			0.408
	Male	51 (30)	48 (34.5)		44 (29.3)	42 (34.7)		31 (26.5)	31 (31.6)	
	Female	119 (70)	91 (65.5)		106 (70.7)	79 (65.3)		86 (73.5)	67 (58.4)	
Nationality				0.174			0.296			0.547
	Nordic	152 (89.4)	127 (91.4)		136 (90.7)	112 (92.6)		104 (88.9)	90 (91.9)	
	Other	2 (1.2)	5 (3.6)		2 (1.3)	4 (3.3)		2 (1.7)	3 (3.1)	
	Missing	16 (9.4)	7 (5)		12 (8)	5 (4.1)		11 (9.4)	5 (5)	
Marital status				0.350			0.527			0.408
	Married	30 (17.6)	37 (26.6)		26 (17.3)	30 (24.7)		15 (12.8)	22 (22.5)	
	Partner	3 (1.8)	3 (2.2)		3 (2)	2 (1.7)		3 (2.5)	2 (2.1)	
	Dating	1 (0.6)	2 (1.4)		1 (0.6)	2 (1.7)		1 (0.8)	2 (2.1)	
	Widow/er	104 (61.2)	73 (52.5)		94 (62.7)	66 (54.5)		74 (63.3)	56 (57.1)	
	Single	19 (11.2)	16 (11.5)		19 (12.7)	15 (12.4)		17 (14.5)	12 (12.2)	
	Missing	13 (7.6)	8 (5.8)		7 (4.7)	6 (5)		7 (6)	4 (4)	
Education				0.061			0.108			0.287
	No education	0 (0)	2 (1.4)		0 (0)	2 (1.6)		0 (0)	2 (2.1)	
	Primary school	58 (34.1)	31 (22.3)		51 (34)	28 (23.1)		42 (35.9)	25 (25.5)	
	Middle school	13 (7.7)	20 (14.4)		11 (7.3)	17 (14.1)		9 (7.7)	13 (13.3)	
	High school	30 (17.7)	26 (18.7)		26 (17.3)	23 (19)		20 (17.1)	19 (19.4)	
	University	15 (8.8)	16 (1.5)		15 (10)	14 (11.6)		13 (11.1)	9 (9.1)	
	Other	1 (0.6)	2 (1.4)		1 (0.7)	1 (0.8)		1 (0.8)	1 (1)	
	Missing	53 (31.2)	42 (30.2)		46 (30.7)	36 (29.8)		32 (27.4)	29 (29.5)	
Arthritis				0.959			0.676			0.664
	Yes	31 (18.2)	25 (18)		29 (19.3)	21 (17.4)		23 (19.7)	17 (17.3)	
	No	138 (81.2)	113 (81.3)		121 (80.7)	100 (82.6)		94 (80.3)	81 (82.7)	
	Missing	1 (0.6)	1 (0.7)							
Diabetes				0.042*			0.076			0.060
	Yes	19 (11.2)	27 (19.4)		18 (12)	24 (19.8)		13 (11.1)	20 (20.4)	
	No	150 (88.2)	111 (79.9)		132 (88)	97 (80.2)		104 (88.9)	78 (79.6)	
	Missing	1 (0.6)	1 (0.7)							
Dementia				< 0.001**			< 0.001**			< 0.001**
	Yes	92 (51.1)	107 (77)		79 (52.7)	93 (76.9)		60 (51.3)	79 (80.6)	
	No	77 (45.3)	31 (22.7)		71 (47.3)	28 (23.1)		57 (48.7)	19 (19.4)	
	Missing	1 (0.6)	1 (0.7)							
Heart diseases				0.846			0.695			0.491
	Yes	112 (65.9)	90 (64.8)		97 (64.7)	81 (66.9)		76 (65)	68 (69.4)	
	No	57 (33.5)	48 (34.5)		53 (35.3)	40 (33.1)		41 (35)	30 (30.6)	
	Missing	1 (0.6)	1 (0.7)							
Kidney diseases				0.061			0.021*			0.020*
	Yes	18 (10.6)	25 (18)		14 (9.3)	23 (19)		10 (8.5)	19 (19.4)	
	No	151 (88.8)	113 (81.3)		136 (90.7)	98 (81)		107 (91.5)	79 (80.6)	
	Missing	1 (0.6)	1 (0.7)							
Lung diseases				0.889			0.547			0.457
	Yes	27 (15.9)	23 (16.6)		22 (14.7)	21 (17.4)		16 (13.7)	17 (17.3)	
	No	141 (82.9)	115 (82.7)		128 (85.3)	100 (82.6)		101 (86.3)	81 (82.7)	
	Missing	2 (1.2)	1 (0.7)							
Autoimmune diseases				0.797			0.986			0.899
	Yes	11 (6.5)	8 (5.8)		10 (6.7)	8 (6.6)		9 (7.7)	8 (8.2)	
	No	158 (92.9)	130 (93.5)		140 (93.3)	113 (93.4)		108 (92.3)	75 (91.8)	
	Missing	1 (0.6)	1 (0.7)							
Other diseases				0.547			0.411			0.975
	Yes	40 (23.5)	37 (26.6)		33 (22)	32 (26.4)		31 (26.5)	26 (26.5)	
	No	128 (75.3)	101 (72.7)		116 (77.3)	89 (73.6)		85 (72.6)	72 (73.5)	
	Missing	2 (1.2)	1 (0.7)		1 (0.7)			1 (0.9)		
Polypharmacy				0.674			0.586			0.742
	Yes	139 (81.8)	116 (83.5)		124 (82.7)	103 (85.1)		96 (82.1)	82 (83.7)	
	No	30 (17.7)	22 (15.8)		26 (17.3)	18 (14.9)		21 (17.9)	16 (16.3)	
	Missing	1 (0.6)	1 (0.7)							

*p < 0.05

**p < 0.001

**Fig. 2:**
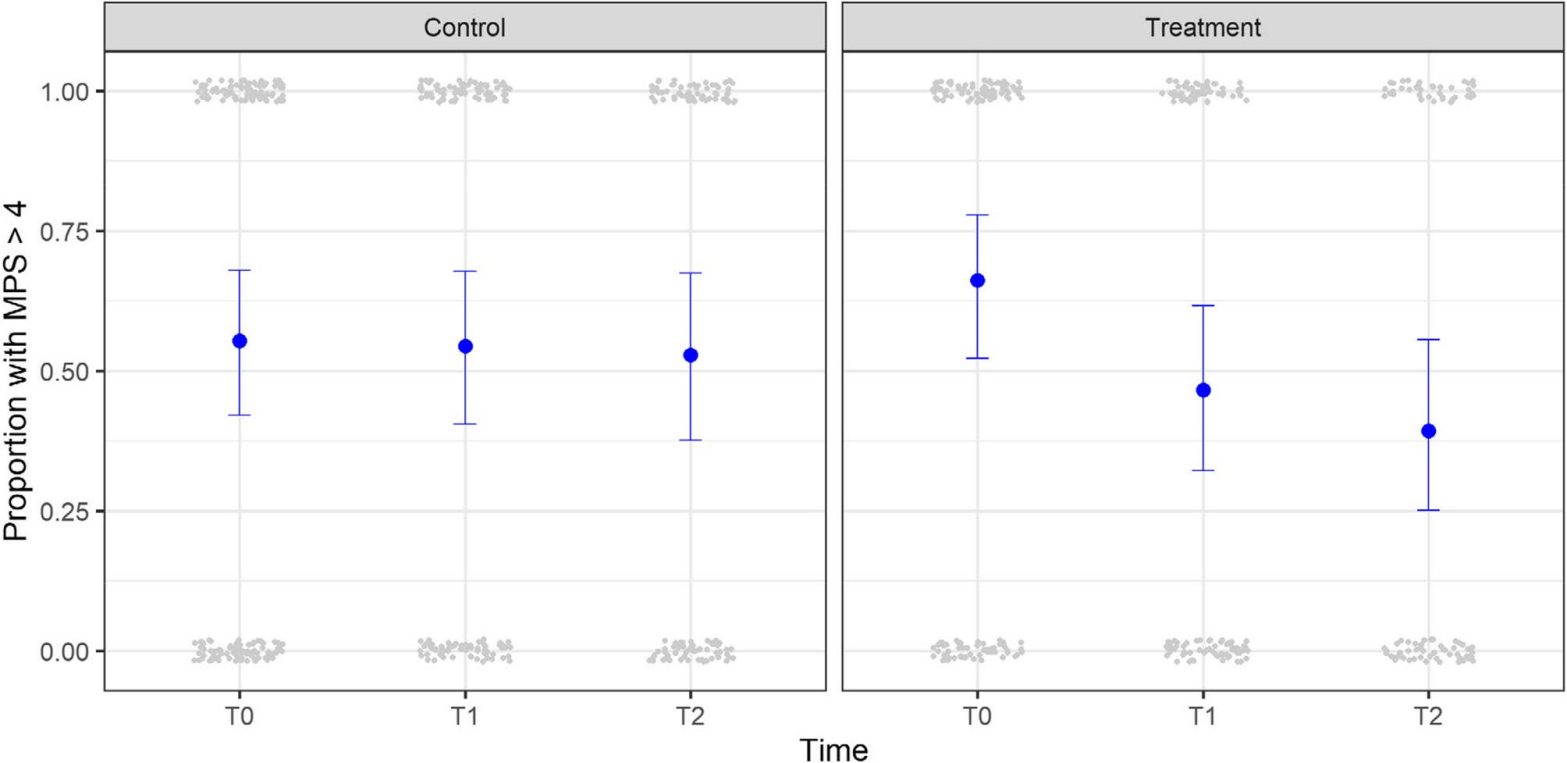
Effect of the SmartJournal intervention on MPS. The small grey data points represent dichotomized raw data. The blue data points and corresponding error bars represent the predicted mean values and 95% confidence intervals within each time and group based on GLMM for binary data, where the random effect factors were participants nested under the nursing home. The time points T0, T1 and T2 represent measurements taken at baseline (T0), and at the 3-month (T1) and 9-month (T2) follow-ups. MPS was based only on natural teeth if possible and otherwise on dentures

Figure 2 shows the results of the GLMM analysis, which examined the differences in the change in MPS values between the intervention and control groups over time. At baseline (T0), no significant difference was observed between the intervention and control groups (*p* = 0.455). Although there was a large difference in effect size between the groups over time (from T0 to T1, from T1 to T2), this difference did not reach statistical significance at T2 (*p* = 0.292).

Notably, within-group analysis comparing baseline data (T0) with follow-up measurements (T1 and T2) revealed that the intervention group tended toward statistical significance at T1 (*p* = 0.097), with a larger reduction in MPS (difference in mean on logit-link scale: −0.768; CI 95%: from − 1.68 to 0.14). At T2, the intervention group demonstrated a statistically significant reduction (*p* = 0.047) in MPS compared with the baseline (difference in mean on logit-link scale: −1.004; CI 95%: from − 2.00 to −0.01). The analysis was conducted using an ITT approach (Fig. 2).

## Discussion

The results from the present study revealed a reduction in the proportion of residents with poor oral hygiene (MPS >4) in both the intervention and control groups, although the difference between groups was not statistically significant. However, the within-group analyses demonstrated a significant improvement among residents in the intervention group after 9 months, illustrating the longevity and sustainability of the intervention. Previous studies have focused primarily on increasing caregiver knowledge and education but have resulted in limited improvements in residents’ oral health [11, 28, 50]. Likewise, no significant group differences in plaque or gingival indices were found in a 6-month follow-up trial that focused on caregivers’ education [32]. Randomised trials that focused on interventions to improve oral hygiene scores among nursing home residents demonstrated either a short-term positive effect on MPS scores [31] or no significant difference in improvement between the intervention and control groups [30]. A longitudinal study assessing the effectiveness of an oral hygiene protocol revealed no effect on dental or denture plaque among institutionalised older adults [29]. This apparent lack of effectiveness in oral care interventions for care-dependent older adults may stem from various challenges [4, 18–21, 51]. A systematic review by Salazar et al. [52] indicated that the feasibility of intervention studies in nursing homes is closely linked to caregivers’ oral health beliefs and attitudes, as well as the cooperation of older people.

Several factors may have contributed to the lack of significant differences between the intervention and control groups in the present study. One possible explanation is that participants in both groups received an oral hygiene package, augmenting the control group. As both groups exhibited reductions in MPS scores, the observed effect size of the intervention was diminished, thereby reducing the measurable difference between the groups [see Additional file 2: Figure S2 and S3]. The baseline data depicted a greater MPS in the intervention group than in the control group, but the difference was not significant between the groups. However, there was a substantially greater incidence of dementia within the intervention group. Given that individuals with dementia are more prone to exhibiting CRB, this population presents additional complexities in clinical management [52]. Individuals with cognitive impairment may perceive dental treatment as invasive or as a violation of personal integrity, further complicating oral health interventions [50]. Furthermore, analysis of individual nursing homes throughout the study revealed that the greatest improvement occurred in a facility assigned to the control group [see Additional file 2: Figure S4]. Other studies have revealed that alternative factors, including the presence of dental professionals or leadership style may encourage subjects to improve the quality of oral care [29–32, 51]. While the results of this study did not reveal a statistically significant difference between the intervention and control groups, the findings remain clinically significant for the target population [53].

### Strengths and limitations

#### Strengths

One of the study’s strengths is the use of a CRT design. CRTs are recommended for evaluating new standards of care, guideline recommendations, or other system-wide changes affecting patient outcomes [36, 54] or when there is a high risk of contamination [36]. The study also demonstrated its robustness by analysing the data utilising both the ITT principle and the PP approach while accounting for fixed and random effects. ITT analysis, which includes all participants as originally allocated, helps minimise attrition bias and reflects real-world effectiveness despite differences in adherence [48]. In contrast, PP analysis focuses only on participants who completed the study, offering insight into the treatment effect under full adherence [48]. This approach aligns with conditions in nursing homes, as all residents would receive treatment upon arrival rather than after a delay.

Furthermore, a minimal number of exclusion criteria have led to a cohort representative of the population of interest, with a high number of residents with dementia [19]. Another strength is the comprehensive calibration before data collection, which provides high reliability and accuracy. Additionally, the use of the MPS as a primary outcome measure is considered a strength as it has excellent IRR and is a valuable tool for long-term oral health assessment in older adults [9, 31, 46].

This study demonstrated a high degree of internal validity, as the randomisation process was concealed from all participants and researchers involved. Additionally, blinding was implemented across three arms, effectively minimising ascertainment bias. Without proper blinding, differential treatment of the groups later in the trial or different assessments of outcomes may result in biased estimates of treatment effects, systematically distorting the results and conclusions of the trial [42, 43]. Given that the individuals included in the study were representative of the population of interest, the strong internal validity enhances the generalisability of the findings (i.e., external validity).

#### Limitations

It is important to acknowledge the study’s limitations. The most evident finding is that the recruitment of participants was managed by nursing home personnel, which may have introduced selection bias and resulted in a cohort with relatively better overall health. While all staff received the same instructions, individual recruitment decisions may have been influenced by subjective factors that are difficult to measure, quantify or control in a real-world pragmatic trial setting. Another key limitation is the loss of statistical power compared with initial power analysis calculations. First, the predicted ICC during the sample size analysis (0.03 or 0.06) was much lower than the actual value (0.23), giving a large design effect. Second, differences in recruitment success resulted in an imbalance in cluster sizes, which further reduced statistical power. This may have contributed to a type-2 error in the analysis [54]. Overall, these limitations could explain the lack of a significant difference between the groups.

### Future implications

The findings of this study, therefore, provide a foundation for future research to further explore the role of digital tools in improving oral hygiene care in institutional settings. Future studies can build these results by addressing the limitations described or exploring implementation across diverse care settings. Additionally, the strengths of this study – such as its real-world application and use of a CRT design, can guide the development of more robust and scalable interventions.

## Conclusion

Although the results of this study did not reveal a statistically significant difference between the groups, participants in the intervention group demonstrated a sustained within-group improvement in oral hygiene. These findings suggest that the SmartJournal tool may support improved oral care practices in nursing home settings, although further research with a larger sample size is needed to confirm its effectiveness.

## Supplementary Information

Supplementary Material 1: Additional file 1. CONSORT checklist for cluster randomised trial.

Supplementary Material 2: Additional file 2: Figure S1. Flow diagram showing recruitment and retention in the trial. Figure S2. Per-protocol analysis of participants with reduction in MPS from T0 to T2. Figure S3. Change in proportion of all participants having unacceptable or poor MPS at T0, T1, and T2. Figure S4. Mean improvement in MPS values from T0 to T2 for each cluster (nursing home).

## Data Availability

The dataset analysed during this study is not publicly available due to the sensitivity of the data but is available from the corresponding author upon reasonable request.

## Abbreviations

CRB: Care-resistant behaviour
SJ: SmartJournal
CRT: Cluster randomised trial
IRR: Inter-rater reliability
ICC_class: Intraclass correlation coefficient
ICC: Intracluster correlation coefficient
MPS: Mucosal-plaque score
MS: Mucosal score
PS: Plaque score
GLMM: Generalised linear mixed-effects model
ITT: Intention-to-treat
PP: Per-protocol
