# Supplementary material for: Evaluating the role of the smartjournal digital intervention in improving oral hygiene among nursing home residents: a cluster randomised trial
Source: BMC Oral Health. 2025 Oct 10;25:1589. doi: 10.1186/s12903-025-06994-0 (PMC12512849; doi:10.1186/s12903-025-06994-0)
Supplement: Supplementary file 1 — Supplementary Material 1: Additional file 1. CONSORT checklist for cluster randomised trial. [file 12903_2025_6994_MOESM1_ESM.pdf]

Additional file 1: CONSORT checklist for cluster randomised trial.

Section/topic	Item No	Checklist item (extension for cluster design)	Reported on page No
**Title and abstract**	1a	Identification as a cluster randomised trial in the title	1
	1b	Structured summary of trial design, methods, results, and conclusions (for specific guidance see CONSORT for abstracts)	1
**Introduction**			
Background and objectives	2a	Scientific background and explanation of rationale (Rationale for using a cluster design)	4
	2b	Specific objectives or hypotheses (Whether objectives pertain to the cluster level, the individual level, or both)	4-5
**Methods**			
Trial design	3a	Description of trial design (such as parallel, factorial) including allocation ratio (Definition of cluster and description of how the design features apply to the clusters)	4
	3b	Important changes to methods after trial commencement (such as eligibility criteria), within reasons	N/A
Participants	4a	Eligibility criteria for participants (eligibility criteria for clusters)	5
	4b	Settings and locations where the data was collected	5
Interventions	5	The interventions for each group with sufficient details to allow replication, including how and when they were actually administered (Whether interventions pertain to the cluster level, the individual level, or both)	8-9
Outcomes	6a	Completely defined prespecified primary and secondar outcome measures, including how and when they were assessed (Whether outcome measures pertain to the cluster level, the individual level or both)	8-9
	6b	Any changes to trial outcomes after the trial commenced, with reason	N/A

Additional file 1: CONSORT checklist for cluster randomised trial (Continued).

Sample size	7a	How sample size was determined (Method of calculation, number of cluster(s) (and whether equal or unequal cluster sizes are assumed), cluster size, a coefficient of intracluster correlation (ICC or *k*), and an indication of its uncertainty	6-7
	7b	When applicable, explanation of any interim analysis and stopping guidelines	N/A
**Randomisation**			
Sequence generation	8a	Methods used to generate the random allocation sequence	4
	8b	Type of randomisation; details of any restriction (such as blocking and block size) (Details of stratification or matching if used)	5
Allocation concealment mechanism	9	Mechanism used to implement the random allocation sequence (such as sequentially numbered containers), describing any steps taken to conceal the sequence until interventions were assigned (Specification that allocation was based on clusters rather than individuals and whether allocation concealment (if any) was at the cluster level, the individual participant level, or both)	4-5
Implementation	10a	Who generated the random allocation sequence, who enrolled clusters, and who assigned clusters to interventions	5
	10b	Mechanism by which individual participants were included in clusters for the purposes of the trial (such as complete enumeration, random sampling)	5-7
	10c	From whom consent was sought (representatives of the cluster, or individual cluster members, or both) and whether consent was sought before or after randomisation	5-7
Blinding	11a	If done, who was blinded after assignment to interventions (for example, participants, care providers, those assessing outcomes) and how	7-8
	11b	If relevant, description of the similarity of interventions	8

Additional file 1: CONSORT checklist for cluster randomised trial (Continued).

Statistical methods	12a	Statistical methods used to compare groups for primary and secondary outcomes (How clustering was taken into account)	9-12
	12b	Methods for additional analyses, such as subgroup analyses and adjusted analyses	9-12
**Results**			
Participant flow (a diagram is strongly recommended)	13a	For each group, the numbers of participants who were randomly assigned, received intended treatment, and were analysed for the primary outcome (For each group, the numbers of clusters that were randomly assigned, received intended treatment, and were analysed for the primary outcome)	7-8, Additional file 2: Figure S1
	13b	For each group, losses and exclusions after randomisation, together with reasons (For each group, losses and exclusions for both clusters and individual cluster members)	Additional file 2: Figure S1
Recruitment	14a	Dates defining the periods of recruitment and follow-up	Additional file 2: Figure S1
	14b	Why the trial ended or was stopped	N/A
Baseline data	15	A table showing baseline demographic and clinical characteristics for each group (Baseline characteristics for the individual and cluster levels as applicable for each group)	10-11
Numbers analysed	16	For each group, number of participants (denominator) included in each analysis and whether the analysis was by original assigned groups (For each group, number of clusters included in each analysis)	Additional file 2: Figure S1
Outcomes and estimation	17a	For each primary and secondary outcome, results for each group, and the estimated effect size and its precision (such as 95% confidence interval) (Results at the individual or cluster level as applicable and a coefficient of intracluster correlation (ICC or *k*) for each primary outcome)	Figure 3, Additional file 2: Figure S4, Figure S5, Figure S6
	17b	For binary outcomes, presentation of both absolute and relative effect sizes is recommended	9-12

Additional file 1: CONSORT checklist for cluster randomised trial (Continued).

Ancillary analyses	18	Results of any other analyses performed, including subgroup analyses and adjusted analyses, distinguishing prespecified from exploratory	Table 2, Figure 3
Harms	19	All important harms or unintended effects in each group (for specific guidance see CONSORT for harms)	N/A
**Discussion**			
Limitations	20	Trial limitations, addressing sources of potential bias, imprecision, and, if relevant, multiplicity of analyses	7-8, 14-15
Generalisability	21	Generalisability (external validity, applicability) of the trial findings (Generalisability to clusters and/or individual participants (as relevant))	13-14
Interpretation	22	Interpretation consistent with results, balancing benefits and harms, and considering other relevant evidence	12-15
**Other information**			
Registration	23	Registration number and name of trial registry	ClinicalTrials.gov (NCT05724043)
Protocol	24	Where the full trial protocol can be accessed, if available	https://www.researchprotocols.org/2023/1/e46926
Funding	25	Sources of funding and other support (such as supply of drugs), role of funders	16
