# Supplementary material for: Evaluating the role of the smartjournal digital intervention in improving oral hygiene among nursing home residents: a cluster randomised trial
Source: BMC Oral Health. 2025 Oct 10;25:1589. doi: 10.1186/s12903-025-06994-0 (PMC12512849; doi:10.1186/s12903-025-06994-0)
Supplement: Supplementary file 2 — Supplementary Material 2: Additional file 2: Figure S1. Flow diagram showing recruitment and retention in the trial. Figure S2. Per-protocol analysis of participants with reduction in MPS from T0 to T2. Figure S3. Change in proportion of all participants having unacceptable or poor MPS at T0, T1, and T2. Figure S4. Mean improvement in MPS values from T0 to T2 for each cluster (nursing home). [file 12903_2025_6994_MOESM2_ESM.pdf]

**Figure S1. Flow diagram showing recruitment and retention in the trial.**

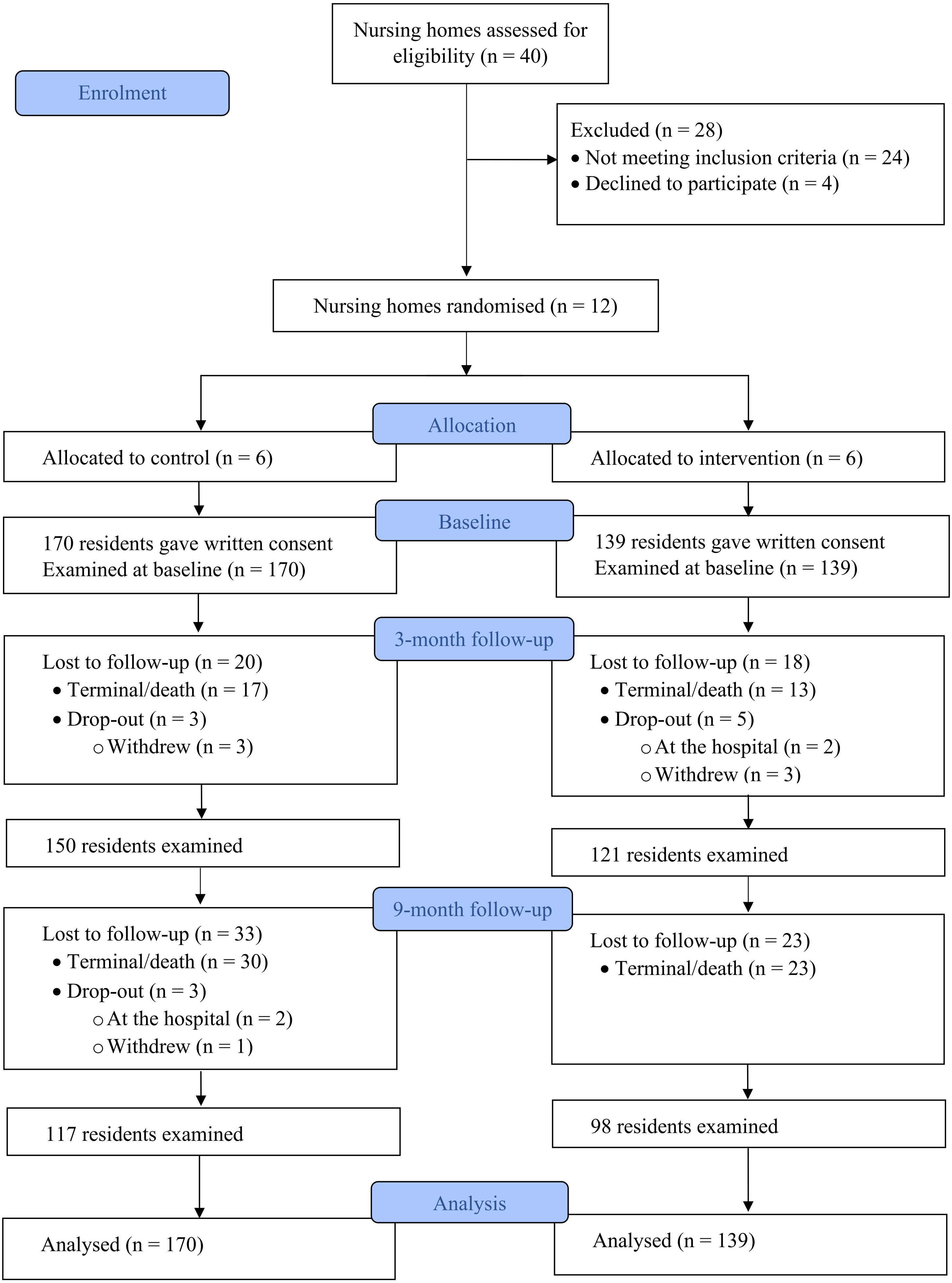

Figure S2 shows the results of a PP analysis using an alternative approach, where individual improvement in MPS between T0 and T2 was used as a binary outcome (reduction = 1; no reduction = 0). This analysis focused on whether a participant experienced a reduction in MPS during the study period. Both groups showed improvement, with a greater proportion of individuals in the intervention group demonstrating a reduction in MPS; however, the between-group difference was not statistically significant (difference in mean on logit-link scale: 0.792; $p = 0.091$; SD = 0.60; CI 95%; from -0.134 to 1.717).

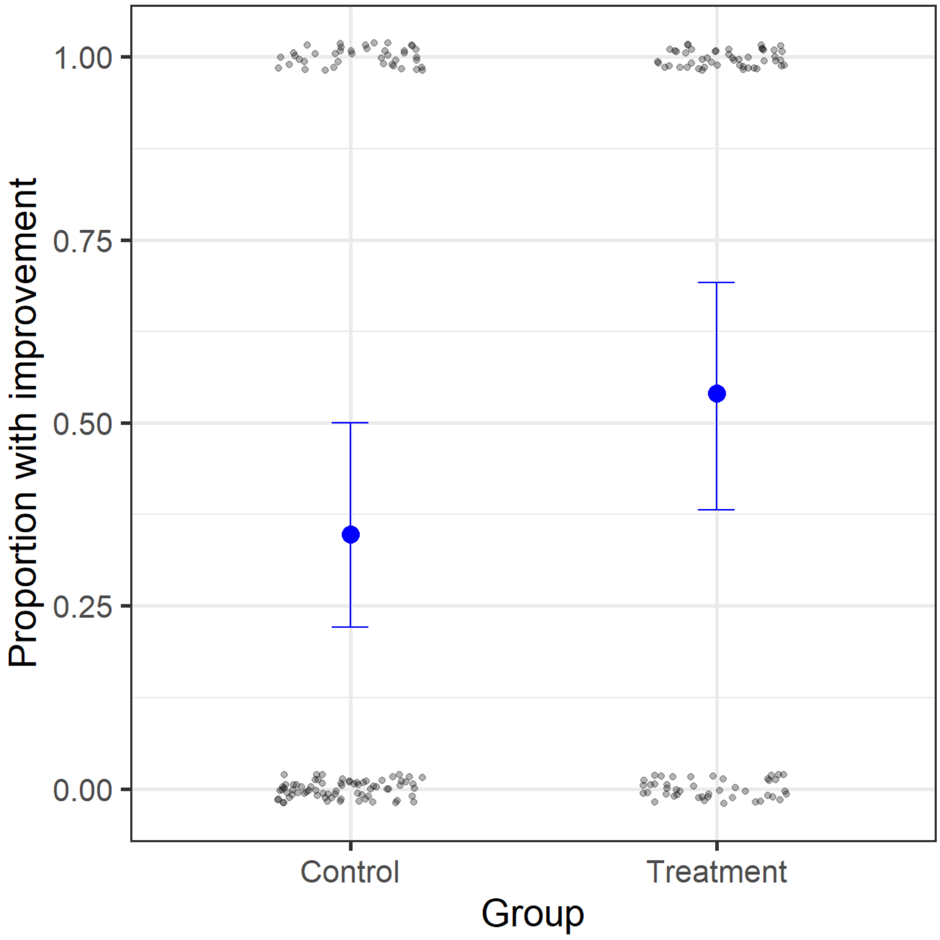


**Figure S2:** Per-protocol analysis of participants with reduction in MPS from T0 to T2. The small grey data points represent individual data where the values on the y-axis represent improvement (1) and no improvement (0) in MPS from measurements taken at baseline and on its last day, respectively. The blue data points and corresponding error bars represent predicted mean values and 95% confidence intervals within each group based on GLMM for binary data, where the random effect factor was the intercept of each nursing home. MPS was based only on natural teeth if possible and otherwise on dentures.

Figure S3 presents the results of MPS measurements across all participants across three time points. When comparing T0 to T1, the difference is not statistically significant, although there

was a trend toward a reduction in proportion of individuals with MPS > 4 at T1 (reduction in mean on logit-link scale: -0.374; $p = 0.079$; CI 95%: from -0.80 to 0.05). At T2, the proportion of individuals with MPS > 4 was significantly reduced compared to T0 (difference in mean on logit-link scale: -0.547; $p = 0.019$; CI 95% from -1.00 to -0.09).

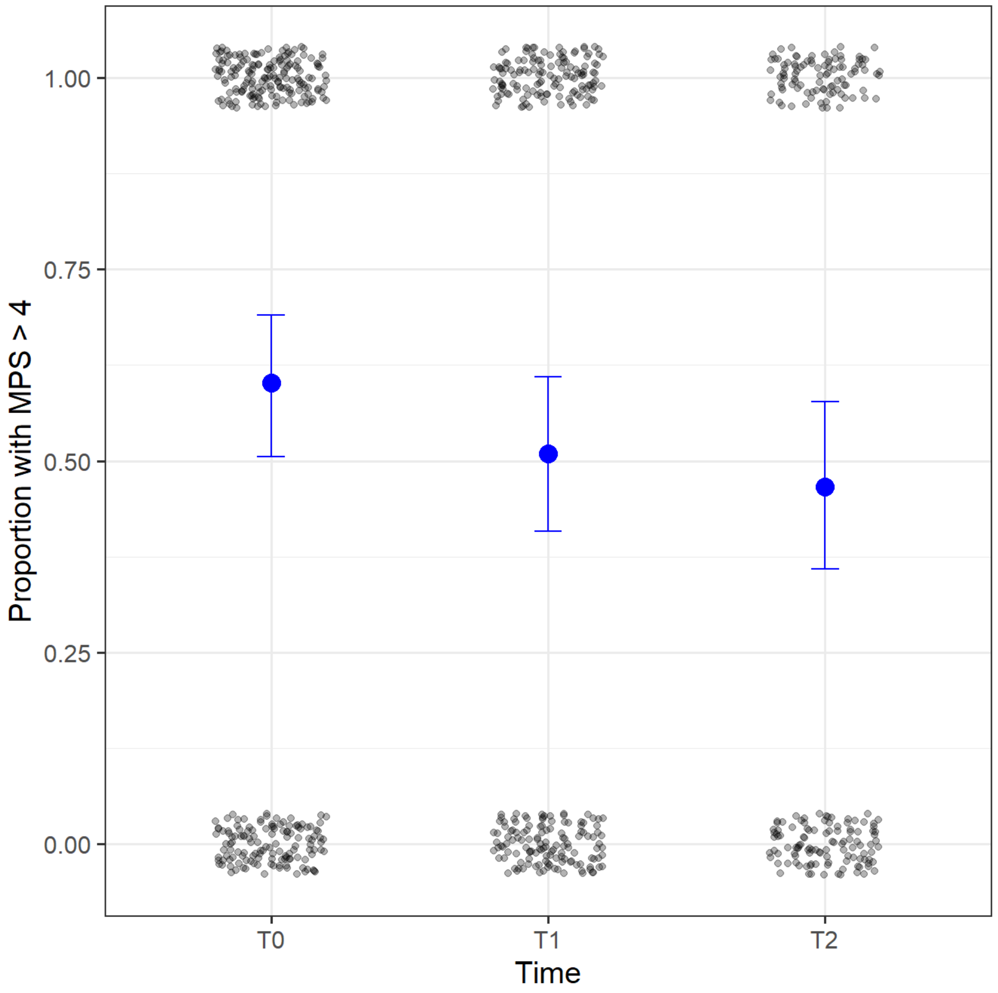


**Figure S3:** Change in proportion of all participants having unacceptable or poor MPS at T0, T1 and T2. The small grey data points represent dichotomized raw data. The blue data points and corresponding error bars represent predicted mean values and 95% confidence intervals within each time based on a GLMM for binary data, where the random effect factors were patient nested under nursing home. Time points T0, T1 and T2 represent measurements at baseline (T0), at 3-month (T1) and 9-month (T2) follow-ups. MPS was based only on natural teeth if possible and otherwise on dentures.

Figure S4 demonstrates a plot with mean improvement at the level of each nursing home from baseline to T2 and the ICC_cluster value. The ICC for nursing home from the model connected to Figure 4 is: ICC = 0.228.

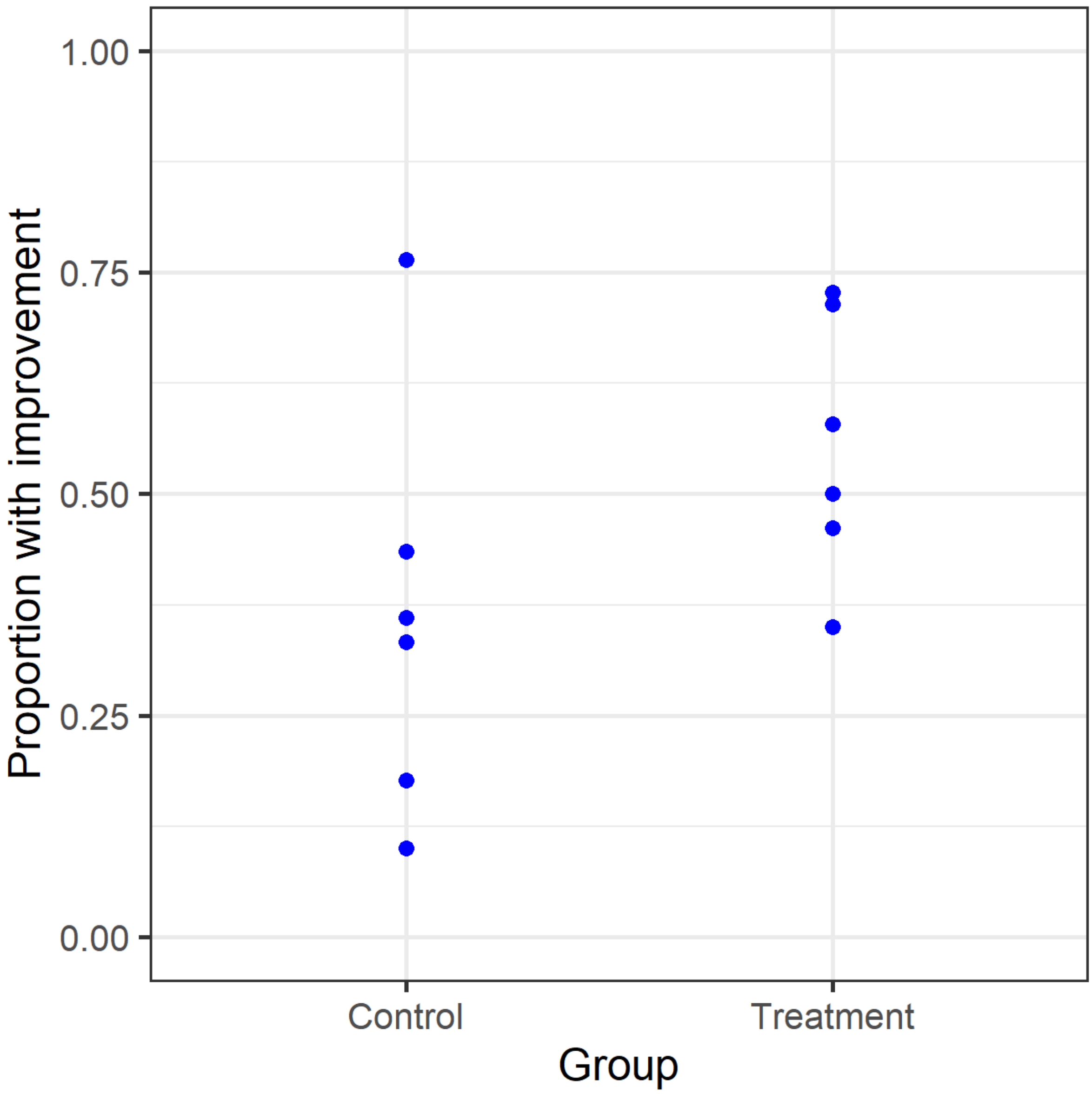


**Figure S4:** Mean improvement in MPS values from T0 to T2 for each cluster (nursing home).
